# Using immunisation caregiver journey interviews to understand and optimise vaccination uptake: lessons from Sierra Leone

**DOI:** 10.1136/bmjgh-2021-005525

**Published:** 2021-05-27

**Authors:** Mohamed F Jalloh, Benjamin Hickler, Lauren E Parmley, Roberta Sutton, Shibani Kulkarni, Anthony Mansaray, Oliver Eleeza, Palak Patel, Elisabeth Wilhelm, Laura Conklin, Adewale Akinjeji, Mame Toure, Brent Wolff, Dimitri Prybylski, Aaron S Wallace, Maria Lahuerta

**Affiliations:** 1 Immunization Systems Branch, Global Immunization Division, Centers for Disease Control and Prevention, Atlanta, Georgia, USA; 2 UNICEF, New York, New York, USA; 3 ICAP at Columbia University, Mailman School of Public Health, New York, New York, USA; 4 Oak Ridge Institute for Science and Education, Oak Ridge, Tennessee, USA; 5 Sierra Leone Country Office, ICAP at Columbia University, Freetown, Sierra Leone; 6 Department of Epidemiology, Mailman School of Public Health, New York, New York, USA

**Keywords:** health systems evaluation, immunisation, child health, qualitative study

## Abstract

Quantitative and qualitative assessments have revealed diverse factors that influence the uptake of childhood immunisation services and shed light on reasons for vaccination delays and refusals. UNICEF and partner organisations developed the Immunisation Caregiver Journey Framework as a novel way to understand caregiver experiences in accessing and receiving immunisation services for children. This framework aims to help immunisation programmes identify vaccination barriers and opportunities to improve vaccination uptake by enhancing the overall caregiver journey in a systems-focused manner, using human-centred design principles. In this paper, we adapt the framework into a flexible qualitative inquiry approach with theoretical guidance from interpretative phenomenology. We draw from the implementation experiences in Sierra Leone to inform methodological guidance on how to design and implement the Immunisation Caregiver Journey Interviews (ICJI) to understand the lived experiences of caregivers as they navigate immunisation services for their children. Practical guidance is provided on sampling techniques, conducting interviews, data management, data analysis and the use of data to inform programmatic actions. When properly implemented, the ICJI approach generates a rich qualitative understanding of how caregivers navigate household and community dynamics, as well as primary healthcare delivery systems. We argue that understanding and improving the caregiver journey will enhance essential immunisation outcomes, such as the completion of the recommended vaccination schedule, timeliness of vaccination visits and reduction in dropouts between vaccine doses.

**Summary box**

- The global burden of childhood diseases has decreased substantially since 1990 largely due to improved access and increased uptake of vaccines offered through the Expanded Programme on Immunisation.
- Despite the availability of vaccination services, caregivers may delay or refuse some or all scheduled vaccines for their children for a variety of reasons, including contextual, individual or vaccine-specific and vaccination-specific influences.
- We developed the Immunisation Caregiver Journey Interviews (ICJI) approach based on the associated Caregiver Journey Framework using interpretative phenomenology as the basis to deeply understand caregiver experiences before, during and after accessing immunisation services for their children.
- The caregiver experience at each stage of the immunisation journey may vary and be influenced by disparate or interrelated factors. Immunisation programmes and independent evaluators alike may find the ICJI approach useful to understand the complexities of vaccination uptake within or across caregiver groups at national or subnational levels.
- A rich, qualitative understanding of the caregiver journey can inform context-specific and culturally responsive interventions to improve vaccination uptake.

## Introduction

Vaccines play a critical role in protecting children from life-threatening and debilitating diseases.^1^ In the last decade alone, more than 1 billion children were vaccinated worldwide through the Expanded Programme on Immunisation.^2^ These efforts have led to the elimination of measles in some geographical regions and near eradication of polio globally.^3 4^ The WHO estimated that in 2019, approximately 85% of all infants globally received three doses of diphtheria–tetanus–pertussis-containing vaccine (DPT3), a standard measure for the performance of an immunisation programme.^5^ Despite high coverage globally, inequities between low-income and middle-income countries (LMICs) and high-income countries persist, though their differences—as measured by DPT3 coverage—declined from approximately 30% in the year 2000 to 15% in 2010.^1^ Inequities in vaccination coverage also exist across and within LMICs,^6^ contributing to undervaccination among those living in conflict-affected places, hard-to-reach areas and urban poor settings^7–11^ and populations with low socioeconomic status.^12^

The proportion of children globally who received DPT3 has stalled at around 85%, despite efforts over the past decade to increase its coverage.^5^ As countries grappled with stagnated vaccination coverage, the COVID-19 pandemic further complicated the situation by inadvertently disrupting the delivery of childhood immunisation services in many countries due to restrictions in population movement and other risk mitigation measures.^13^ However, even before the COVID-19 pandemic, approximately 20 million children missed one or more scheduled vaccine doses annually.^14^

Current evidence suggests that even when vaccination services are available, caregivers may delay or refuse some or all vaccines for their children for a variety of reasons, including contextual, individual or vaccine-specific and vaccination-specific influences.^15^ This phenomenon has been termed ‘vaccine hesitancy’ by the WHO and has been listed among the top 10 threats to health globally.^16^ Beyond reasons related to vaccine hesitancy, hard-to-reach populations may also be undervaccinated, which contributes to inequities in vaccination coverage. Hard-to-reach populations include groups that face ‘barriers to vaccination due to geography by distance or terrain, transient or nomadic movement, healthcare provider discrimination, lack of healthcare provider recommendations, inadequate vaccination systems, war and conflict, home births or other home-bound mobility limitations or legal restrictions’.^17^

## Towards an experience-driven approach

Expectancy-value theories such as the Health Belief Model have largely informed past assessments of vaccination attitudes, intentions and behaviours.^18–26^ These expectancy-value theories do not adequately account for the dynamic role of caregivers’ repeated experiences in shaping their vaccination behaviours. Recognising these limitations, various international partner organisations, under the leadership of UNICEF, developed the Immunisation Caregiver Journey Framework^27^ as a novel way to contextualise and understand how caregivers and families navigate childhood immunisation services, especially in LMIC settings. The framework aims to help identify vaccination barriers and leverage opportunities to improve vaccination uptake in a systems-focused manner using principles of human-centred design.^27^

In operationalising the framework in the context of qualitative inquiry, phenomenology provides a suitable methodological basis^28 29^ for examining the lived experiences of caregivers as they access childhood immunisation services in diverse settings. Focusing on specific events and the related experiences allows caregivers to provide rich information that thoroughly describes the immunisation journey in the context of both health systems and social conditions.^30^ The caregiver experience may vary and be influenced by disparate or interrelated factors at each stage of the immunisation journey. Holistic exploration of the journey helps to provide a nuanced understanding of how caregivers navigate household dynamics; community norms, processes and structures; and healthcare delivery systems. Therefore, rigorous qualitative inquiry of the caregiver journey is needed to explore the range of diverse experiences and events occurring before, during and after accessing immunisation services. Here, we describe the development of a qualitative approach for conducting Immunisation Caregiver Journey Interviews (ICJI), using interpretative phenomenology as the basis to deeply understand caregiver experiences and the potential influences on vaccination outcomes.

## Intended use and audience

The ICJI approach provides a practical methodology to qualitatively explore and critically examine caregiver experiences when accessing childhood immunisation services, with a contextual focus on LMIC settings. Adaptions of the ICJI may also be appropriate in high-income country settings. The approach is intended to be used to conduct face-to-face in-depth interviews^31–33^ with caregivers of children who are eligible for immunisation services (or catch-up doses), and the child has received one or more vaccines through a fixed-post strategy at a vaccination site (eg, health facility). It may also be adapted for use in focus group discussions. Given that the approach explores caregiver experiences in accessing and receiving immunisation services, its current format is not readily appropriate for use with caregivers who have *never* accessed immunisation services for their children or whose children only received vaccines via door-to-door campaigns (eg, polio vaccination campaigns).

Depending on the context and needs, the ICJI approach can be used as a stand-alone assessment or incorporated into other planned data collection efforts. In table 1, we have summarised the strengths, challenges and considerations when integrating the ICJI approach into existing data collection opportunities within immunisation programmes such as the Review of the Expanded Programme on Immunisation (EPI Reviews),^34^ Tailoring Immunisation Programmes,^35^ New Vaccine Post-Introduction Evaluation^36^ and Reaching Every District.^37^ Standalone ICJI assessments may not always be feasible for various reasons—for instance, when resources are not available for a standalone assessment. Even when resources are available, integration may be more advantageous where the emphasis is on ensuring that the ICJI data can be triangulated and used alongside other immunisation systems assessment data such as during EPI Reviews. Moreover, the ICJI approach may be adapted for use in efforts aiming to understand the impact of the COVID-19 pandemic (or other future health emergencies) on childhood immunisation.

**Table 1:** Summary of opportunities for integrating the Immunisation Caregiver Journey Interviews (ICJI) into existing data collection efforts in immunisation programmes

Why integrate?	What are the key strengths?	What are the potential challenges?	What are some key considerations for success?
**Review of the Expanded Programme on Immunisation (EPI Reviews):** comprehensive assessments of national and subnational immunisation programmes^34^			
ICJI may inform issues and barriers related to service delivery and vaccination uptake from the caregiver perspective	Ability to get information systematically every 3–5 years across multiple countries Strong participation from diverse stakeholders across the immunisation programme	Lack of skilled interviewers and qualitative analysts as part of the EPI Review teams Difficult to turn around the data quickly to identify preliminary findings within the EPI Review timeframe (usually within 1–2 weeks)	Link and partner with local academic institutions Create a pool of subregional experts to provide support Initiate the ICJI approach a month ahead of the EPI Review Focus on descriptive narratives to identify key themes rapidly Long-term local capacity building for qualitative expertise
**Tailoring Immunisation Programmes (TIP)**: conducted in multiple phases among low-uptake groups to identify vaccination barriers and develop targeted solutions to increase uptake^48^			
ICJI can be used in the phases pertaining to identifying barriers and facilitators related to childhood immunisation.	Time needed for TIP is favourable to accommodating ICJI in the initial phases that include situation analysis and additional research ICJI can provide information to subsequent phases of the TIP implementation	More concerted efforts required to integrate the results into the immunisation programme Given the fluidity of the local environment, the results may lose their relevance if too much time is taken to conduct the assessment and analyse the data TIP has mainly been implemented in European region countries thus far	Include key individuals and institutions involved in the immunisation programme into relevant aspects of the assessment to allow for the results to be used by the immunisation Link and partner with local academic institutions to identify key researchers/staff that can be involved in this assessment The TIP framework needs adaptation for use outside of European countries
**New Vaccine Post-Introduction Evaluation (PIE**): an evaluation method for assessing the impact of introducing a new vaccine or vaccine dose in the immunisation schedule^36^			
ICJI can be integrated in PIE data collection efforts with caregivers to understand their experiences with a new vaccine or vaccine dose	Use of mixed methods Strong participation from diverse stakeholders	More concerted efforts required to effectively incorporate qualitative inquiry into standardised PIE Short turnaround time for data collection and analysis	Questions would need to be adapted for a specific new vaccine Focus the analysis on descriptive narratives to identify key themes rapidly
**Reaching Every District (RED**): aims to strengthen immunisation systems by improving planning, managing available resources, service delivery and routine monitoring^37^			
ICJI can be embedded into the participatory social mapping in RED to identify barriers through the caregiver experiences	ICJI can help to understand context-specific and population-specific issues affecting low vaccination uptake	Concerted efforts likely required to effectively incorporate qualitative inquiry into RED social mapping activity	ICJI guide will potentially require substantial adaptations for the specific vulnerable/undervaccinated subpopulations Focus on using descriptive narratives to identify key themes rapidly that can be used to complete RED tools on mapping barriers in the specific community

## Development and refinement of the interview guide

We drew from the interpretative phenomenological tradition in qualitative research to inform our development of the interview guide for the ICJI. We initially drafted broad, open-ended questions under three temporal areas related to caregiver experiences when accessing childhood immunisation services—before, during and after accessing immunisation services at a health facility. We developed open-ended questions and probes to understand (1) decision-making and preparation, (2) making the journey to the vaccination site, (3) experiences at the vaccination site, (4) postvaccination experiences and (5) intentions to return for scheduled vaccination visits. In designing the Sierra Leone ICJI assessment, we held a stakeholder engagement meeting where we received feedback from diverse representatives from the Sierra Leone Ministry of Health, civil society partners and non-governmental organisations supporting immunisation efforts in Sierra Leone. The feedback we received from the stakeholder engagement informed our initial development of the ICJI interview guide, which was subsequently piloted in Freetown, Sierra Leone, with a convenience sample of four caregivers that were recruited from the immunisation clinic in a paediatric hospital. We used feedback from caregivers to refine and expand the interview guide—for instance, we added questions and probes to explore how immunisation services are promoted in communities. Also, in the revised guide, additional probes were included to get more nuanced information regarding the relationship and interactions between caregivers and health workers. We piloted the revised guide once again with a separate convenience sample of four caregivers who were recruited from urban communities in Freetown, Sierra Leone. This process led to the finalisation of the questions and probes in the ICJI guide (table 2). During implementation, the interviewers had opportunities to do ‘on-the-spot’ probing for additional information based on the specific experiences of the individual caregivers. In addition to the interview guide, we have provided sample consent statement and interview cover sheet (online supplemental material).

10.1136/bmjgh-2021-005525.supp1 Supplementary data

**Table 2:** List of domains, questions and probes in the Immunisation Caregiver Journey Interviews guide

Domain	Questions	Probes
Decision-making and preparation	1) What were the things you took into consideration when thinking about taking your child to be vaccinated?	a) How did you know when to take the child? b) Who played a role in deciding to take the child for vaccination? c) Who made the final decision to take the child for vaccination? d) What did you anticipate or expect regarding the visit based on your experience from prior visits or things that people may have told you?
	2) Once the decision was made to take the child for vaccination, please tell me about how you prepared for the visit.	a) What did you do to remember the date? b) How did you manage the visit with work or other duties? c) What did you have to do to get to the health facility? d) Did someone help you to get there or go with you? Please explain. e) What did you have to do to get into the health facility?
Making the journey	3) Please tell us about the health facility where you went for the last vaccination visit, and what was the journey like to get there?	a) How far was it, and how did you get there? b) What was the journey like to get there? Was it easy or challenging to get there and for what reasons? c) How many times had you been to this facility in the past? d) What do you think about the time it usually took before vaccination staff attended to your child? e) Were there any costs associated with the immunisation services? If so, please explain: Did you have to pay anyone to get your child vaccinated? If so, how much and to whom? Did you have to provide any goods or materials to get your child vaccinated? If so, what did you provide and to whom?
Experiences during vaccination visit	4) Please describe the overall experience during your last vaccination visit.	a) What did you like about the visit and for what reasons? b) What did you NOT like about the visit and for what reasons? c) How did this experience make you want to return or NOT return for other vaccinations? d) In the future, what can the staff at the vaccination site do to improve the experience for you and other caregivers?
	5) Please describe your interactions with healthcare workers, including the vaccinator, during your last vaccination visit at the health facility.	a) How would you describe your experience when interacting with the vaccination staff? b) What do you think about how the vaccination staff that interacted with you? How did the interaction make you feel? What did you like about the interaction? What did you NOT like about the interaction? c) How does this experience make you want to return or NOT return? Please tell us more. d) In the future, what can vaccination staff do to improve the experience for you?
	6) Please describe your interactions with other caregivers while you were waiting to get your children vaccinated.	a) What kind of interactions did you have with other caregivers? b) What were caregivers saying? What were they doing? c) Was there something that was done/said by other caregivers that may have encouraged or discouraged you to bring your child back for other vaccination? Please help us understand.
Postvaccination experiences	7) Please tell us about what happened after the visit once you returned to your community.	a) Did anyone ask you questions? If so, who asked about your experience? What did they ask? How did you reply? If not, what would you say if people asked about your experience? b) How were other caregivers talking about their experiences (if applicable)? c) In general, what do people in your community say about vaccination? d) In general, what do people in your household say about vaccination?
	8) Please tell us about how the last visit may be similar or different from your other prior visits for this child or your other children.	a) How did your experiences change or remained the same over time? Please help us understand. b) What experiences were similar? Please help us understand. c) What experiences were different? Please help us understand.
	9) Have you, or someone you know, ever had a child that experienced any adverse events after being vaccinated?	a) Please describe exactly what happened (signs, symptoms, timeline, etc.) and the circumstances. b) Who did what for the child? What was the outcome? c) How did you feel about this experience? d) What did you say to others about this experience? e) How do other people in the household or community feel about the situation?
Intentions to return	10) When is your child’s next visit for vaccination? *You can check the Child Health Card if you have it (if respondent can’t read, check the date and tell her; then, ask the following questions*)	a) Do you plan to attend that next scheduled vaccination visit for this child? b) If YES, what are the key reasons why you would return to the vaccination site? c) If NO, what are the key reasons why you would NOT return to the vaccination site?
Demand promotion	11) How is immunisation promoted in your community?	a) What information are you provided, how frequent and by whom? b) What do you think about the information? c) What questions do you have about immunisation, if any? d) Who do you usually trust to talk to you about the health of your child? Who do you trust the most to talk to you about immunisation? Who do you trust the least to talk to you about immunisation? e) What do you think about how immunisation services are promoted in your community? How do you think the promotion activities for immunisation can be improved

## Sampling techniques and data collection

When implementing the ICJI assessment in a specific area, we recommend purposive sampling of at least 12 eligible caregivers and continuing until data saturation is reached based on the concept of *qualitative information power* assessed against the study aim, sample specificity, established theory, quality of dialogue and analysis strategy.^38^ For instance, instead of interviewing 12 caregivers, a larger sample of caregivers may be necessary to achieve information power if the assessment is adapted to compare vaccination experiences between multiple population subgroups. According to the concept of information power, sampling burden is generally reduced when the aim is narrow (vs broad), the sample is targeting a specific group (vs multiple groups with varying attributes of interest), established theory is applied to inform the assessment (vs no application of theory), the quality of dialogue is strong (vs weak) and case-based analysis is performed (vs cross-case analysis). These attributes combined should be critically appraised before the initiation of the assessment, periodically during data collection and again after data collection to ascertain information power.

To be eligible, (1) caregivers should have at least one child who is eligible for immunisation services or eligible to receive catch-up doses previously missed, and (2) the child has received one or more vaccines through a fixed-post strategy at a vaccination site. Concerted efforts should be made to obtain a sample of caregivers with diverse backgrounds so that contextualised differences and similarities in caregiver experiences can be captured. To help maximise variation in the sample, quota sampling may be leveraged to ensure having a minimum number of certain groups of caregivers or attributes of interest. We have provided an example of a target sample of caregivers, which may be adapted for different contexts (table 3).

**Table 3:** Example of a target distribution of caregivers to interview, ICJI approach

Proximity	Child vaccination status	Number of caregivers
<5 miles from health facility	Fully up to date on all doses	3
	Delayed or missed >1 dose	3
>5 miles from health facility	Fully up to date on all doses	3
	Delayed or missed >1 dose	3
		12

ICJI, Immunisation Caregiver Journey Interview.

Caregivers may be recruited using snowball sampling whereby four eligible caregivers are identified first, one from each of the four categories of eligible caregivers outlined in table 3; then, ask each of the initial four caregivers to refer you to other similar, eligible caregivers in the community. Alternatively, a review of the health facility immunisation registers may be used to identify children who meet the criteria outlined in table 3. If caregiver selection is based on register review, it is important to avoid the involvement of health workers in the selection because they may bias towards caregivers who they know are likely to provide certain desirable responses. Use of the ICJI approach is also appropriate to embed within a qualitative longitudinal design that recruits one or more cohorts of eligible caregivers to conduct repeat interviews at different time points.^39^

One or more data collection teams may be needed depending on the sample design, desired turnaround time to collect the data and other logistical considerations. We recommend that each team be composed of two members—an interviewer and a dedicated note-taker, which is customary in qualitative investigations. The interviewer should primarily be responsible for asking the questions in the guide while the note-taker takes copious notes of the conversation. With the caregiver’s permission, the interview may be audio-recorded to allow for a more accurate capturing of the conversation. The interviewer and note-taker should conduct a 20–30 min debriefing session at the end of each interview.^40^

In Sierra Leone, our initial strategy was to recruit caregivers through health facilities on immunisation clinic days. In piloting this strategy, we learnt that it was difficult to locate previously identified caregivers in their communities. This was particularly evident in slum settlements where distinctive address systems are non-existent. We subsequently shifted to a community-based recruitment strategy with on-the-ground support from community health workers (CHWs) who helped us to identify eligible caregivers. After identifying some initial caregivers with the help of CHWs, we used a snowball sampling technique wherein caregivers assisted the data collection team in identifying other eligible caregivers in their communities. An important lesson learnt is that CHWs play a very important role in identifying and locating caregivers and should be leverage in future ICJI assessments other similar settings. In most instances, CHWs had to accompany data collectors to the homes of caregivers, which helped the teams to establish rapport with the caregivers. Moreover, we found it useful to have the flexibility to schedule interviews outside of normal working hours to avoid conflicts with caregivers’ livelihood activities. Some caregivers needed the endorsement of their husband/partner or male head of household to participate in the assessment. At times, a male decision-maker was the custodian of the child’s vaccination card, which was needed to verify the child’s vaccination status. Respectfully and sensitively navigating these cultural norms was important for successful recruitment and implementation of the interviews.

## Data analysis and interpretation

Each interview conducted as per the ICJI approach should yield the following: (1) interview notes; (2) debrief notes; (3) audio recording, where feasible; and (4) transcripts. No matter the theoretical underpinning, qualitative data analysis begins with immersion into the data by thoroughly reading and rereading transcripts and notes.^33^ In implementing the ICJI in Sierra Leone, we conducted both within-case and cross-case analyses. Consistent with interpretative phenomenological analysis (IPA),^41^ we dedicated considerable amount of time analysing the transcript from each caregiver and developing individual narrative profiles with thick descriptions, verbatim quotes and iterative interpretations of the documented experiences. We inductively coded all transcripts with the aid of NVivo V.12 software. Additionally, we used a qualitative content analysis approach to facilitate the grouping of the inductive codes into categories using Microsoft Excel.^42^ Finally, in developing cross-cutting themes, we iteratively interpreted findings from the within-case and cross-case analyses to deeply understand convergence and divergence in the immunisation caregiver journeys.

We learnt from this process that although there are theoretic tensions between IPA and qualitative content analysis, the two can be successfully combined wherein content analysis is used in a non-theoretical fashion (ie, only for inductive coding and categorisation) and interpretative phenomenology provides the theoretical basis for exploring, analysing and interpreting the caregiver experiences using within-case and cross-case analyses. For instance, we only identified one caregiver who routinely refused all vaccines for her child. The within-case analysis of this caregiver was used to provide a rich account of why some caregivers may actively refuse all vaccines for their children. In the final thematic write-up, we captured and interpreted both the thick descriptions of individual experiences and their shared experiences to give meaning to the immunisation caregiver journey in low-resource urban communities of Sierra Leone.

## Reflexivity and making sense of the data

Reflexivity is an important attribute of qualitative inquiry.^43^ In analysing the ICJI data from Sierra Leone, the two primary analysts (one woman and one man) brought their own lifeworld experiences and perspectives to the inductive analysis and interpretation. Both analysts and their contributors had experience working in global immunisation with diverse experiences conducting social and behavioural assessments to understand childhood vaccination experiences and behaviours in numerous countries in West Africa. On the one hand, having had prior experience in childhood immunisation work within West Africa, including Sierra Leone, allowed the team to draw from these experiences in interpreting the data. On the other hand, such experience had the potential to create blind spots. During the early stage of the inductive coding and analysis of transcripts, the analysts shared three of the initial coded transcripts with a third analyst that did not have any experience working in Sierra Leone or sub-Saharan Africa to ‘blindly’ code the transcripts so that we get an ‘outsider’ perspective. For instance, the ‘outside’ analyst more pronouncedly identified the complex role of gender dynamics on the caregiver journey, which the two analyst also identified but may have been more inclined to take for granted given their preconceived sociocultural knowledge of gender roles and cultural norms in Sierra Leone.

In analysing the pilot data from Sierra Leone, we identified numerous areas in the transcripts that needed additional follow-up probing. For instance, we observed that time was considered as a commodity by most of the caregivers, and many of them consistently complained about the ‘waste of time’ due to prolonged delays at vaccination sites. Further probing would have provided more nuanced understanding of this notion of time as a commodity. In addition, although we had some anecdotal awareness of the obstacles faced by caregivers when accessing childhood immunisation services, we were surprised by the extent of the compounded practical constraints and logistical challenges that caregivers had to overcome in taking their children for vaccination services. Another surprising observation was the complexity surrounding monetary exchange between caregivers and health workers. Based on our prior experiences in Sierra Leone and LMICs elsewhere and knowledge of the literature, we were aware that some health workers may request informal payments from caregivers in exchange for vaccinating their children,^44^ though heavily discouraged by official policies aiming to provide free health services at no charge.^45^ After analysing the data, we learnt that although many caregivers begrudgingly gave money to health workers, there were instances when they also willingly did so to show their appreciation. Based on what we have learnt from Sierra Leone, future ICJI assessments need to dig deeper into these complexities around the relationship between caregivers and health workers.

## Informing programmatic actions

Following the ICJI assessment in Sierra Leone, there were several programmatic actions taken after we shared the results with the Ministry of Health and stakeholders in the immunisation programme including implementing partners and donors. Insights from the assessment informed the design of joint in-service training that covered various topics including interpersonal communication and community outreach for over 400 health staff involved in childhood immunisation in urban catchment areas in Western Area, Sierra Leone. In addition, the findings were used to advocate for and support a more active role of CHWs in the tracking of children who have missed scheduled immunisation in urban communities, including in urban slums.

## Conclusion

The ICJI approach and the emerging insights from the Sierra Leone assessment provide methodological and programmatic considerations for ongoing and future efforts aiming to understand and improve childhood immunisation services and outcomes.^46 47^ Immunisation programmes may be able to improve vaccination outcomes by gaining a deep understanding of the caregiver journey and using those insights to address complex barriers while harnessing opportunities at the individual, community and health systems levels.

## Data Availability

All data relevant to the study are included in the article or uploaded as supplementary information.
