# Supplementary material for: Using immunisation caregiver journey interviews to understand and optimise vaccination uptake: lessons from Sierra Leone
Source: BMJ Glob Health. 2021 May 27;6(5):e005525. doi: 10.1136/bmjgh-2021-005525 (PMC8162096; doi:10.1136/bmjgh-2021-005525)
Supplement: Supplementary data [file bmjgh-2021-005525supp001.pdf]

Supplementary materials

Example of Consent Statement

Good morning/afternoon/evening. My name is ___________________. I am working with ___________________. Thank you for agreeing for me to interview you today. I am part of a team that is working to understand facilitators, barriers, and ways to improve the uptake of immunization services among children less than three years of age in ________________ district or sub-district.

We would like to learn about your experiences accessing and using these services. Specifically, we want to learn about factors that help you or make it more difficult to use these services. We are interested in getting your ideas on how to improve these services.

In this interview, there are no right or wrong answers. We are interested in getting your thoughts and opinions. We want to understand your experiences in your own words. While we ask that you speak candidly, you do not have to answer questions you do not feel comfortable discussing.

As a reminder, we will keep private the information that you share during this interview. With your permission, this interview may be audio-recorded. We use the audio recording to transcribe the interview and ensure that we accurately capture your responses. We will label the audio recording, transcripts, and information sheet you are your participant identification number. We will replace your name and other details that would identify you or anyone that you mention during the interview with a general term. We will never link your responses to you in any written reports.

I will be asking most of the questions today. My co-facilitator may ask some follow-up questions and will be taking notes to go along with the recording. As we go through the interview, please let me know if there any questions you would rather not answer or if you need a break.

Do you have any questions at this point?

Do you agree to participate in this interview?

Yes → Continue

No → STOP

Do we have your permission to audio recorded this interview?

Yes → Continue with recording

No → DO NOT record; only take notes

<u>Interviewer Attestation of Informed Consent</u>

I, _________________, acknowledge that I have obtained informed consent for this interview.

Signature: __________________ Date: __________________

Example of Interview Cover Sheet

Date	|___||___| / |___||___| / |___||___|*(dd/mm/yy)*
Respondent ID	|___||___|
Age of child	|___||___| *years* |___||___| *months*
Child vaccination status (based on age)	1. Received *all scheduled vaccines on time*<br>2. Delayed or missed *one or more doses*
Area of residence	1. Urban [___ *slum*]<br>2. Rural [___ *hard-to-reach*]
District & community	________________|________________
Proximity to nearest health facility	|___||___| *miles*
Relationship to child	1. Parent<br>2. Grandparent<br>3. Sibling<br>4. Other (specify________________)
Respondent sex	1. Male<br>2. Female
Respondent age	|___||___| *years*
Respondent education	1. No education<br>2. Primary<br>3. Secondary +
Facilitator name (first, last)	
Start Time	|___||___| : |___||___|*(hour/min)*
End Time	|___||___| : |___||___|*(hour/min)*
Name of electronic file	
